# Metal‐Organic Framework‐Templated Synthesis of 2D Co_3_S_4_@Co‐BPDC Nanosheets for High‐Performance Electrochemical Energy Storage

**DOI:** 10.1002/smtd.70839

**Published:** 2026-07-14

**Authors:** Zhiwei Xu, Qian Liu, Lin Zhou, Mengru Huang, Yanping Li, Long Kuai, Chuanjian Zhou, Lijie Zhang, Wai‐Yeung Wong

**Affiliations:** ^1^ Anhui Province Key Laboratory of Functional Coordinated Complexes for Materials Chemistry and Application, School of Chemical and Environmental Engineering Anhui Polytechnic University Wuhu P.R. China; ^2^ School of Material Science and Engineering and State Key Laboratory of Coatings for Advanced Equipment Shandong University Jinan P.R. China; ^3^ Zhejiang International Joint Laboratory of Optical Functional Materials Wenzhou University Wenzhou P.R. China; ^4^ Department of Applied Biology and Chemical Technology and Research Institute For Smart Energy The Hong Kong Polytechnic University Kowloon Hong Kong P.R. China; ^5^ The Hong Kong Polytechnic University Shenzhen Research Institute Shenzhen P.R. China

**Keywords:** 2D composite nanosheet, electrochemistry, in situ hydrothermal growth, supercapacitor, templated synthesis

## Abstract

Two‐dimensional (2D) metal‐organic framework (MOF) nanosheets have been widely applied in energy storage. However, single‐component MOF nanosheets face limitations in large‐scale applications due to their poor conductivity and insufficient stability. Therefore, the development of MOF‐based composite nanosheets with enhanced conductivity and stability through the incorporation of a second component has attracted widespread attention. In this work, a series of petal‐like 2D **Co_3_S_4_@Co‐BPDC** composite nanosheets were synthesized by a MOF‐templated synthesis method. Combining the high specific surface area and porous properties of MOF with the excellent stability and conductivity advantages of metal sulfides, the **Co_3_S_4_@Co‐BPDC‐3** electrode without binders and conductive additives demonstrates an exceptional capacity of 2589.2 F·g^−1^ at 1.0 A·g^−1^, coupled with a good cycling stability of 90.5% capacity retention after 1000 cycles. The coin‐type asymmetric supercapacitor (ASC) device displays a maximum energy density of 110.4 Wh·kg^−1^ at a power density of 1699.9 W·kg^−1^, retaining 92.1% capacitance after 10 000 cycles. Furthermore, the flexible ASC device delivers 45.1 Wh·kg^−1^ energy density at 1500.3 W·kg^−1^ with 81.8% retention after 5000 cycles, maintaining 94.4% capacity even under 180° bending. This work established a MOF‐templated synthesis method for 2D composite nanosheets, enabling a rational design strategy toward high‐performance 2D energy storage materials.

## Graphical abstract

A coin‐type asymmetric supercapacitor (ASC) device was fabricated with 2D **Co_3_S_4_@Co‐BPDC** nanosheets, which displayed a maximum energy density of 110.4 Wh·kg^−1^ at a power density of 1699.9 W·kg^−1^, retaining 92.1% capacitance after 10 000 cycles. Furthermore, the flexible ASC device was also prepared and delivered a 45.1 Wh·kg^−1^ energy density at 1500.3 W·kg^−1^ with 81.8% retention after 5000 cycles, maintaining 94.4% capacity even under 180° bending. This work established a MOF‐templated synthesis method for 2D composite nanosheets, enabling a rational design strategy toward high‐performance 2D energy storage materials.

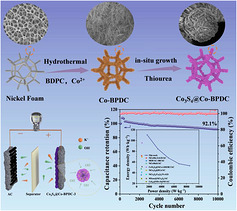

## 1 Introduction

In response to the growing demands of renewable energy integration and portable electronics, advanced energy storage materials must simultaneously achieve high efficiency, sustainability, and scalability [1]. In this context, among the most common energy storage devices (such as supercapacitors, lithium‐ion batteries, etc.), supercapacitors have garnered significant attention and are widely used in high‐power machinery and portable smart wearable devices due to their exceptional power density, long cycle life, and rapid charge/discharge capabilities [2, 3, 4]. However, due to their narrow operating voltage window and insufficient energy density, the large‐scale application of supercapacitors still faces significant limitations, making the development of electrode materials particularly crucial for enhancing their energy storage capabilities [5, 6].

Since the advent of graphene [7], two‐dimensional (2D) nanosheets with polymeric structures such as silicene and black phosphorus have garnered significant attention in fields like electronics, batteries, and supercapacitors, thus driving research and development in related 2D nanosheet materials [8]. Owing to their unique electronic structure and distinct physical advantages, these 2D nanosheets feature ideal ordered nanopores and interlayer spaces for ion insertion/extraction, which facilitate maintaining ion diffusion pathways and accelerating electron transfer, making them promising candidates for supercapacitors with superior electrochemical performance. Consequently, designing novel 2D materials for energy storage and conversion has emerged as a key strategic approach for their future applications [9].

Representative 2D nanosheets primarily include 2D metal‐organic frameworks (MOFs) and covalent organic frameworks (COFs) [10, 11]. The layered structure of 2D MOFs significantly increases the specific surface area, providing more active sites for ion adsorption and charge storage [12, 13]. Additionally, 2D MOFs serve as more favorable precursors for synthesizing high‐performance energy storage materials through processes like sulfidation and carbonization [14]. The ultrathin nature of 2D structures enables closer contact between metal centers and organic ligands with electrolytes, thereby enhancing redox reaction efficiency and demonstrating outstanding pseudocapacitive behavior in supercapacitors [15]. Furthermore, the layered architecture shortens electron and ion transport pathways, reduces charge recombination probability, optimizes charge transport kinetics, and exhibits remarkable advantages in supercapacitor applications [16]. Nevertheless, due to the poor conductivity and structural instability, the development of MOFs in the field of supercapacitors has been limited [17].

To address these challenges, researchers have integrated MOFs with other functional compounds to develop MOF‐derived composite materials. These hybrids not only preserve the intrinsic advantages of individual components but also generate synergistic effects, resulting in novel physicochemical properties unattainable by any single constituent alone [18, 19]. MOFs‐derived composite materials, such as metal oxides, sulfides, layered double hydroxides (LDHs), and carbon derivatives, have higher conductivity, larger specific surface area, and more stable performance, making them ideal candidates for charge storage in supercapacitors [20]. For example, Pang and colleagues developed a homogeneous nitrogen‐doped graphene carbon/metal‐organic framework (N‐GLC/MOF) composite material. Benefiting from the synergistic interaction between these components, the composite achieved a high specific capacitance of 470.18 F·g^−1^ at 1 A·g^−1^, maintained a Coulombic efficiency of 95.04% over 5500 cycles at 5 A·g^−1^, and exhibited a capacitance retention rate approaching 90% in the N‐GLC/MOF‐74//AC (AC = active carbon) configuration [21]. Li and co‐workers fabricated a 3D cross‐linked VO_x_/V_2_CT_x_‐MXene‐x (VO_x_/MXene‐x) composite and a 3D self‐supporting TMA‐V_2_CT_x_/CoV‐LDH/NF composite via a one‐step solvothermal method, both of which exhibit excellent conductivity and high redox reaction activity [22, 23]. Among various composite materials, metal sulfides demonstrate a suite of advantageous properties that render them highly promising for electrochemical applications, including high specific capacity, abundant redox sites, and enhanced conductivity [24]. These characteristics make them ideal candidates for integration with MOFs. For instance, Hu and co‐workers sulfurized Co‐NiBTC as precursor to obtain Co‐NiS_2_/C, which exhibited a large capacitance of 1080 F·g^−1^ at 1 A·g^−1^ and still maintained 89.2% stability after 8000 cycles at 3 A·g^−1^. The Co‐NiS_2_/C//AC asymmetric supercapacitor device exhibited an energy density of 36 Wh·kg^−1^ at a power density of 1271.5 W·kg^−1^ [25]. Wang and co‐workers prepared a NiCo‐LDH precursor using ZIF‐67 as raw material through the sulfurization of NiCo‐LDH@HOS. The fiber electrode exhibits a high specific surface area capacitance of 1100 mF·cm^−2^ at 2 mV·s^−1^. In addition, the flexible all‐solid‐state fiber‐shaped asymmetric supercapacitor based on NiCo‐LDH@HOS//AC exhibited an energy density of 44.37 µWh·cm^−2^ with a power density of 7442.31 µW·cm^−2^ [26]. Although metal sulfides possess high electrical conductivity and abundant pseudocapacitance, they are often plagued by poor cycling stability and severe volume expansion. Employing MOFs as self‐sacrificial templates or precursors represents a highly efficient strategy for constructing sulfide nanomaterials with well‐defined structures and superior performance [27]. This strategy not only preserves the inherent advantage of high electronic conductivity but also significantly enhances porosity, rendering the resulting materials highly suitable for high‐performance supercapacitors. Furthermore, by employing MOFs as self‐sacrificial templates for the synthesis of sulfide@MOF composites, the effect of varying thiourea addition amount on the morphology, structure, and properties of the resulting materials remains to be further investigated.

Based on the aforementioned considerations, the 2D **Co_3_S_4_@Co‐BPDC** nanosheets have been successfully synthesized via MOF‐templated synthesis. First, **Co‐BPDC** MOF was fabricated by Co^2+^ ion and BDPC (4,4'‐biphenyldicarboxylic acid) on nickel foam (NF) through a simple hydrothermal method. After that, **Co‐BPDC** was used as a sacrificial template to be sulfurized with thiourea by an in situ hydrothermal growth to give the petal‐like 2D **Co_3_S_4_@Co‐BPDC** composite nanosheets. The benefit of this MOF‐templated synthesis method is that the metal sulfides (Co_3_S_4_) derived from **Co‐BPDC** MOF can inherit the characteristics of large surface area and high porosity of the original MOF, as well as the excellent conductivity and mechanical stability of metal sulfides. Meanwhile, the in situ growth method eliminates the need for binders and conductive additives during electrode fabrication. It is exciting to observe that during the process of adjusting thiourea dosage, the originally templated nanospine‐like **Co‐BDPC** underwent a morphological transformation, forming petal‐like composite nanostructures. The resulting **Co_3_S_4_@Co‐BPDC‐3** electrode delivered an exceptional specific capacitance of 2589.2 F·g^−1^ at 1.0 A·g^−1^, coupled with an outstanding cycling stability (90.5% capacitance retention after 1000 cycles). Density functional theory (DFT) calculations were employed to construct relevant models, confirming that sulfurization significantly enhances OH* adsorption capability and electron transfer kinetics. To assess the practical applicability, both the coin‐type asymmetric supercapacitor (ASC) device and flexible ASC device incorporating gel electrolytes were assembled. The former demonstrated a maximum energy density of 110.4 Wh·kg^−1^ at a power density of 1699.9 W·kg^−1^ and maintained 92.1% capacity retention after 10 000 cycles, while the latter gave a maximum energy density of 45.1 Wh·kg^−1^ at a power density of 1500.3 W·kg^−1^ and maintained 81.8% capacity retention after 5000 cycles. The flexible ASC device retained 94.4% specific capacity retention even when subjected to 180° bending without structural failure.

## 2 Results and Discussion

### 2.1 Synthesis of **Co_3_S_4_@Co‐BPDC** nanosheets

0.580 g of Co(NO_3_)_2_·6H_2_O (2 mmol) and 0.484 g of BPDC (2 mmol) were thoroughly dissolved in 60 mL of degassed DMF solution, then 5 mL of water and 5 mL of ethanol were added dropwise into the solution. Afterwards, the blend was transferred to a 100 mL PTFE‐lined stainless‐steel autoclave, and the mixed solution was sonicated for 30 min. A precleaned and thoroughly dried nickel foam (NF, 1.5 × 3.7 cm) was then immersed in the prepared solution. The autoclave was heated to 160°C and maintained at that temperature for 12 h. After cooling, the **Co‐BPDC**‐loaded NF was cleaned with deionized (D.I.) water and ethanol alternately for three times and then dried in a vacuum oven at 60°C for 12 h to obtain **Co‐BPDC**.

A suitable proportion of thiourea was added into 40 mL of D.I. water, and then the mixture was stirred and sonicated until the thiourea dissolved. The resulting solution was transferred to the 100 mL high‐pressure reaction vessel. After that, the as‐prepared **Co‐BPDC** was added to the above solution which was then heated at 160°C for 12 h. After cooling, the sample was taken out and washed alternately with ethanol and D.I. water three times; then, the desired nanosheets **Co_3_S_4_@Co‐BPDC** were obtained by in situ growth on NF. According to the amount of thiourea added (0.005 g, 0.010 g, 0.050 g, 0.100 g), the products were marked as **Co_3_S_4_@Co‐BPDC‐1**, **Co_3_S_4_@Co‐BPDC‐2**, **Co_3_S_4_@Co‐BPDC‐3,** and **Co_3_S_4_@Co‐BPDC‐4**, respectively.

### 2.2 Morphology of **Co_3_S_4_@Co‐BPDC**

**FIGURE 1:**
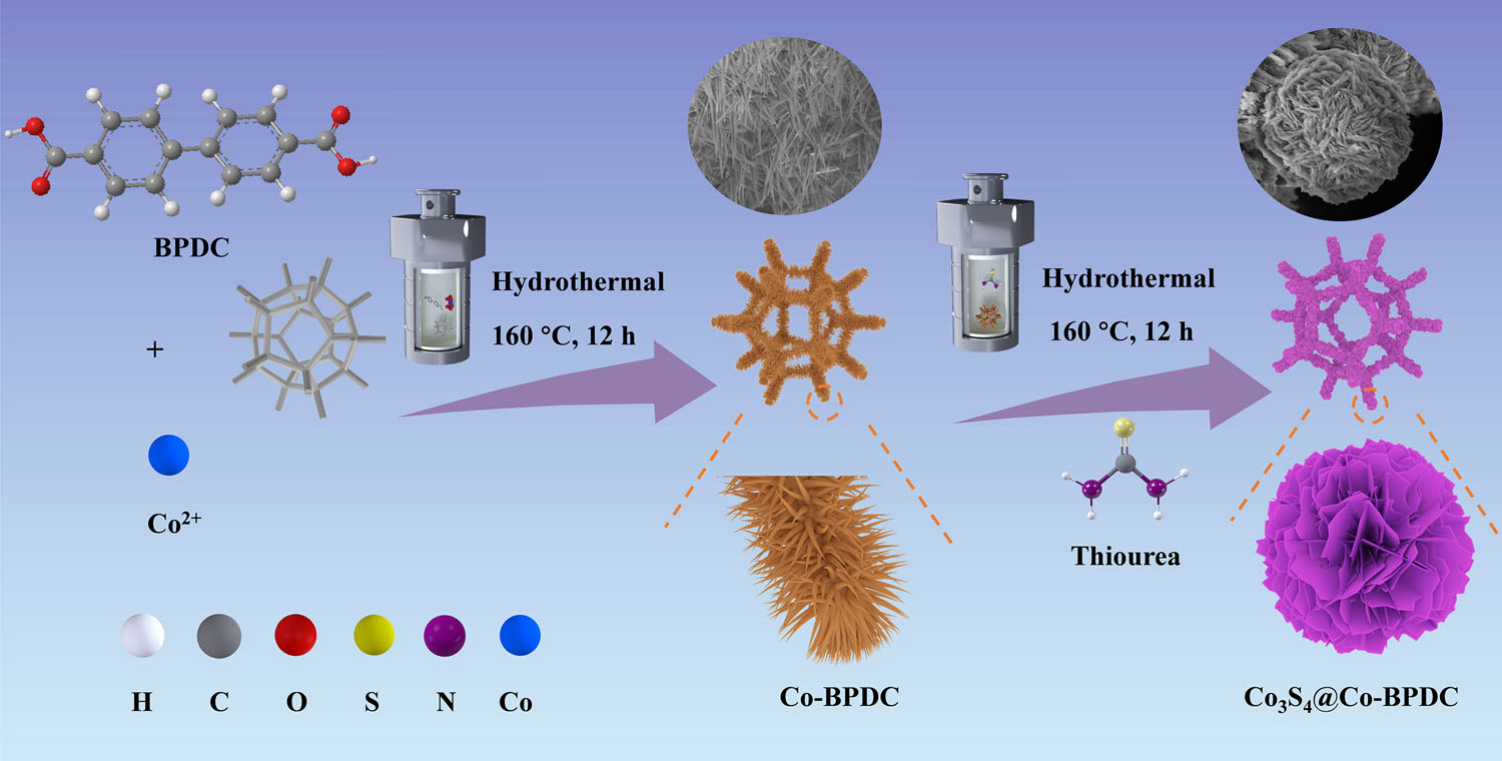
Schematic diagram of the synthetic method of **Co‐BPDC** and **Co_3_S_4_@Co‐BPDC**.

**Co_3_S_4_@Co‐BPDC** nanosheets with petal‐shaped structures have been fabricated via simplified two‐step hydrothermal methods as schematically illustrated in Figure 1. The process begins with the hydrothermal synthesis of **Co‐BPDC**, and through the coordination reaction between Co^2+^ ions and the organic ligand (BPDC), nanospine‐like **Co‐BPDC** uniformly grows on the NF substrate. Then, sulfur sources (thiourea) are introduced under controlled hydrothermal conditions. Interestingly, as the reaction progresses, the initially nanospine‐shaped structure evolves into **Co_3_S_4_@Co‐BPDC** nanosheets, which exhibit a distinct petal‐like morphology. Here, various amounts of thiourea were utilized to prepare a series of **Co_3_S_4_@Co‐BPDC** nanosheets, demonstrating the superiority of the structure and electrochemical properties (vide infra).

**FIGURE 2:**
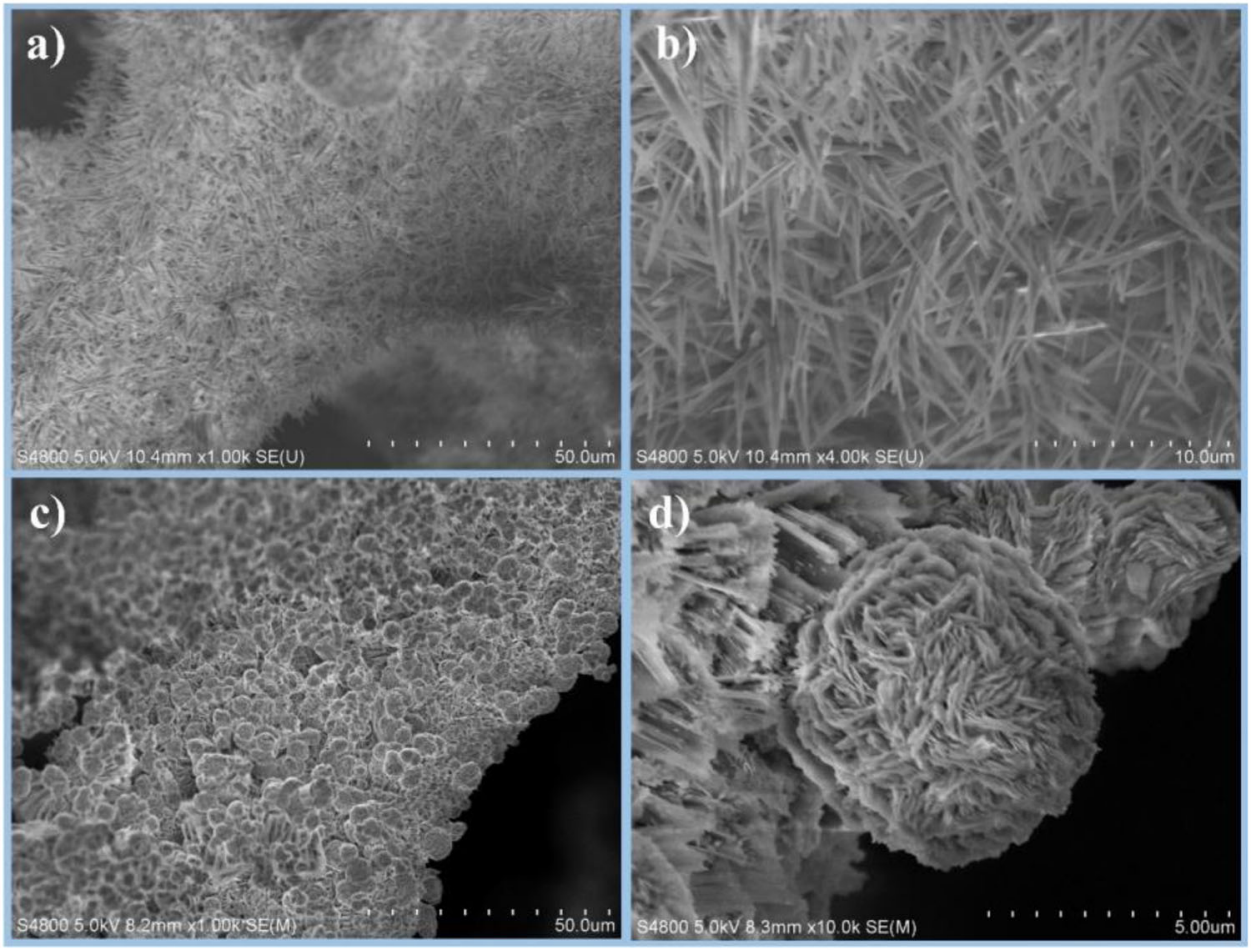
Different magnifications of SEM images of (a, b) **Co‐BPDC** and (c, d) **Co_3_S_4_@Co‐BPDC‐3**.

The morphology of as‐synthesized samples was characterized by scanning electron microscopy (SEM) (Figure 2 and Figure S1). **Co‐BPDC** precursor grows on NF in nanospine morphology with a diameter of approximately 0.4 µm (Figure 2a,b). The SEM images of **Co_3_S_4_@Co‐BPDC‐3** in Figure 2c,d reveal a nanoflower structure with a tightly arranged 2D petal‐like morphology. This lamellar morphology could enhance the concentration of the active sites, while the interlayer gaps facilitate electrolyte penetration and ion transport, thereby improving the electrochemical performance and mechanical stability of **Co_3_S_4_@Co‐BPDC‐3**. Additionally, the continuous crystal structure that exists along the 2D plane provides a high‐speed conduction path for electrons [28]. Figure S1 displays the SEM images of **Co_3_S_4_@Co‐BPDC‐1**, **Co_3_S_4_@Co‐BPDC‐2,** and **Co_3_S_4_@Co‐BPDC‐4**, respectively. Morphological analysis revealed that as the amount of thiourea added increased, the morphology of **Co_3_S_4_@Co‐BPDC** changed. When the amount of thiourea additive is 0.005 g, **Co‐BPDC** nanospines gradually undergo sulfurization and aggregation, and when the amount of thiourea additive is increased to 0.010 g, nanospines have gradually begun to transform into nanosheets and aggregate to nanoflower‐like structures. As the amount of thiourea added is increased to 0.050 g, the nanoflower gradually becomes regular and orderly and evenly distributed on NF, but with the gradual increase of the thiourea added to 0.100 g, the nanoflower‐like structures gradually disappear while the morphology becomes disordered. These observations suggest that excessive thiourea disrupts the petal‐like nanoarchitecture, potentially compromising the electrochemical performance through structural degradation.

**FIGURE 3:**
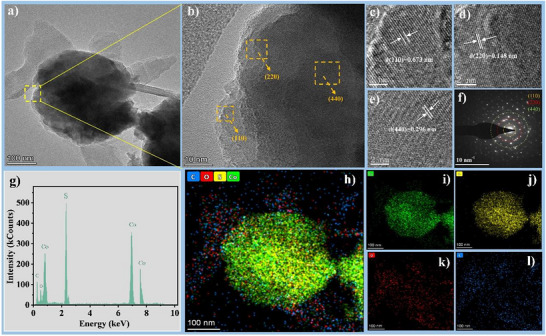
(a) TEM image of **Co_3_S_4_@Co‐BPDC‐3**. (b–e) HRTEM images of **Co_3_S_4_@Co‐BPDC‐3**. (f) SAED image of **Co_3_S_4_@Co‐BPDC‐3**. (g–l) EDS mapping images of **Co_3_S_4_@Co‐BPDC‐3**.

The nanoflower structure with a tightly arranged 2D petal‐like morphology in **Co_3_S_4_@Co‐BPDC‐3** has been further revealed by transmission electron microscopy (TEM) image (Figure 3a). The high‐resolution transmission electron microscopy (HRTEM) images (Figure 3b–e) and selected area electron diffraction (SAED) pattern (Figure 3f) confirm the polycrystalline nature for **Co_3_S_4_@Co‐BPDC‐3**. Specifically, the measured lattice spacing of 0.673 nm corresponds to the (110) crystallographic plane of **Co‐BPDC**, while 0.148 and 0.296 nm are aligned with the (220) and (440) planes of Co_3_S_4_, respectively [29]. This dual‐phase configuration demonstrates successful Co_3_S_4_ generation within the preserved MOF matrix. In addition, the corresponding energy‐dispersive spectroscopy (EDS) elemental mapping images of **Co_3_S_4_@Co‐BPDC‐3** reveal the homogeneity in the distribution of C, O, S, and Co on **Co_3_S_4_@Co‐BPDC‐3** as proposed (Figure 3g–l). The above structural features, including the retained MOF frameworks, exposed Co_3_S_4_ active facets, and anisotropic charge transport pathways, lay a solid foundation for enhancing the electrochemical performance.

**FIGURE 4:**
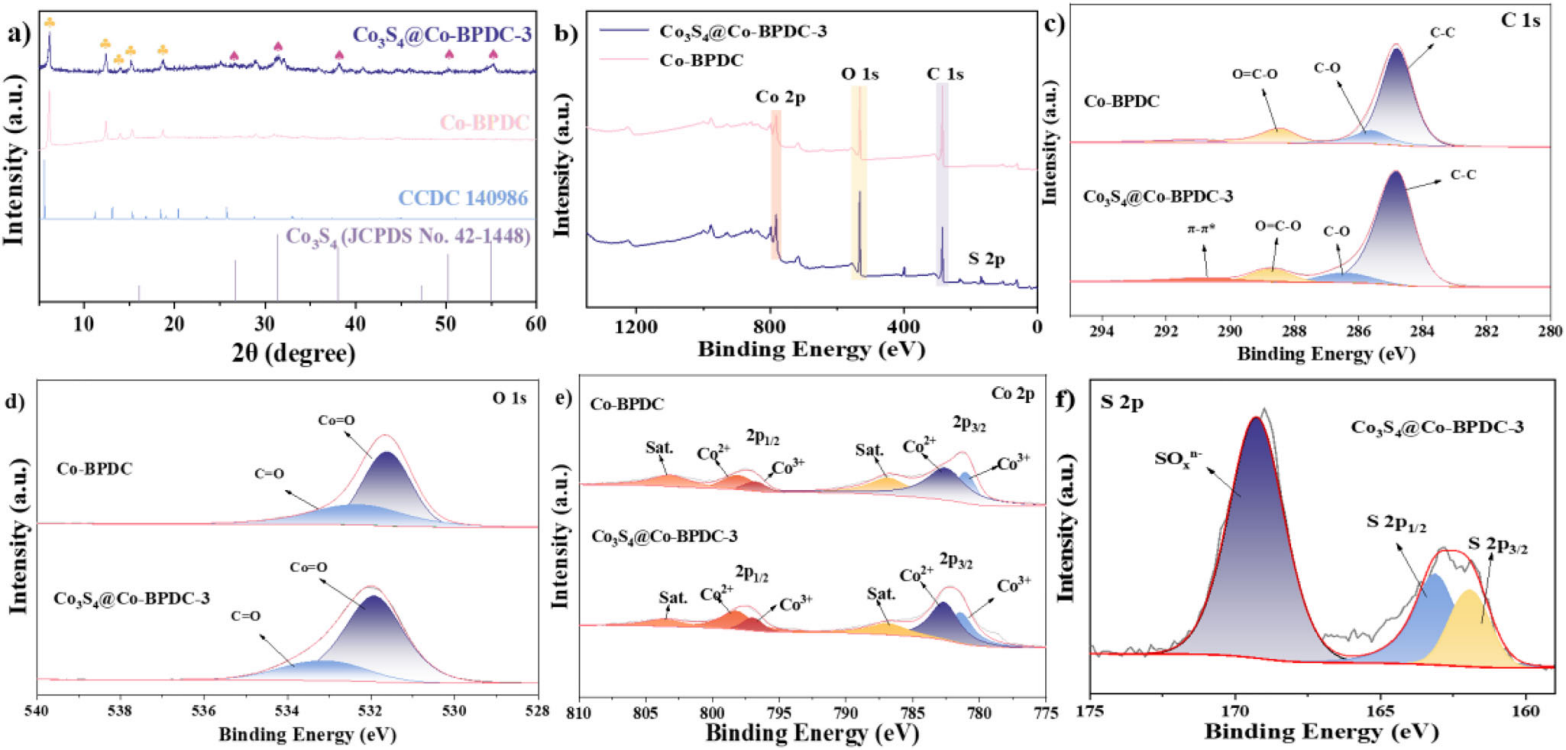
(a) XRD patterns of **Co‐BDPC** nanospines and **Co_3_S_4_@Co‐BPDC‐3** nanosheets. (b) Survey XPS spectrum of **Co‐BDPC** nanospines and **Co_3_S_4_@Co‐BPDC‐3** nanosheets. High‐resolution XPS spectra of (c) C 1s; (d) O 1s; (e) Co 2p and (f) S 2p, respectively.

X‐ray diffraction (XRD) and x‐ray photoelectron spectroscopy (XPS) analyses were sequentially performed to investigate the crystal structures and chemical bonding states and valence configurations of the as‐synthesized samples (Figure 4 and Figure S2). To eliminate interference from the NF substrate, the samples were ultrasonically peeled off from their surface. As shown in Figure 4a, the reflections detected at 5.9°, 12.9°, 14.5°, 15.3°, and 18.3° correspond to the (020), (110), (130), (021), and (041) lattice planes in the **Co‐BPDC** structure (CCDC 140986) [30]. After the introduction of thiourea, characteristic diffraction peaks of metal sulfides (marked with red ♠‌) appeared in **Co_3_S_4_@Co‐BPDC**, while residual **Co‐BPDC** diffraction signals (marked with orange ♣) indicate incomplete conversion. The diffraction pattern exhibits prominent peaks at 26.2°, 31.4°, 38.3°, 50.4°, and 55.3°, which are indexed to the (220), (311), (400), (511), and (440) crystallographic planes of cubic Co_3_S_4_ (PDF No. 42–1448). This further confirms that Co^2+^ reacts with thiourea to form Co_3_S_4_ [26]. The thiourea dosage critically determines the properties of **Co_3_S_4_@Co‐BPDC**. An optimal amount (0.050 g, **Co_3_S_4_@Co‐BPDC‐3**) yields the sharpest diffraction peaks, reflecting the highest crystallinity, and a specific surface area approximately 3.5 times that of pristine **Co‐BPDC** (vide infra). Insufficient thiourea (0.005 g, **Co_3_S_4_@Co‐BPDC‐1**) leads to weak peaks from incomplete sulfidation, while an excess amount (0.100 g, **Co_3_S_4_@Co‐BPDC‐4**) causes peak broadening and missing reflections due to structural distortion and pore blockage, which degrades performance [31].

As depicted in Figure 4b and Table S1, the full XPS spectra confirmed the presence of C, O, and Co in precursor **Co‐BPDC**, while sulfur was detected in the thiourea‐modified **Co_3_S_4_@Co‐BPDC‐3**, which is consistent with the EDS data and elemental quantification. As shown in Figure 4c, the high‐resolution comparative spectrum of C 1s for the two as‐synthesized samples showed no significant change in the carbon form. The characteristic peaks at 284.8, 286.1, 288.6, and 290.8 eV correspond to the C─C bond, C─O bond, O═C─O bond, and π–π* bond, respectively. The slight binding energy shifts indicate the interactions between Co_3_S_4_ and **Co‐BPDC**. O 1s spectra revealed negligible differences between **Co‐BPDC** and **Co_3_S_4_@Co‐BPDC‐3**, in which the peak at 531.9 eV corresponds to Co═O bonds, while 533.1 eV relates to C═O bonds (Figure 4d) [32]. As shown in Figure 4f, the high‐resolution S 2p spectrum of **Co_3_S_4_@Co‐BPDC‐3** exhibits doublet peaks at 163.31 eV and 164.73 eV for 2p_3/2_ and 2p_1/2_, respectively. The observed binding energies correspond to Co‐S bonds, matching the documented spectral signatures of metal sulfides [33]. A distinct peak at 169.17 eV can be assigned to SO_x_^n−^ species, presumably generated by surface oxidation upon air contact [34]. The high‐resolution XPS spectra of Co 2p are depicted in Figure 4e. The spin‐orbit doublet at 796.9 and 781.3 eV correspond to Co 2p_1/2_ and Co 2p_3/2_, which are attributed to Co^3+^, respectively. Another spin‐orbit doublet at 798.2 and 782.6 eV is related to Co^2+^, which corresponds to Co 2p_1/2_ and Co 2p_3/2,_ respectively. In addition, the accompanying satellite peaks at 803.4 and 786.7 eV are characteristic of Co 2p transitions [35]. These spectral signatures collectively corroborate successful material synthesis. Additionally, according to the Fourier‐transform infrared (FT‐IR) spectroscopic analysis in Figure S3, a characteristic peak attributed to the Co─O bond emerges at 665.3 cm^−1^ post cobalt ion coordination. Following sulfur source modification, a distinct Co─S bond signal appears at 320.2 cm^−1^ [36]. These spectroscopic signatures collectively consolidate the successful synthesis of MOF‐derived sulfides.

The porous characteristics of **Co‐BPDC** and **Co_3_S_4_@Co‐BPDC‐3** were systematically investigated through nitrogen adsorption‐desorption experiments at 77 K (Figure S4). Brunauer‐Emmett‐Teller (BET) analysis revealed the specific surface areas of **Co‐BPDC** nanospines and **Co_3_S_4_@Co‐BPDC‐3** nanosheets were 52.6 and 183.2 m^2^·g^−1^, respectively, confirming a threefold increase in specific surface area after sulfurization (Figure S4a). This enhancement originates from structural reorganization along with the generation of abundant active sites, thus facilitating efficient utilization of electrochemically active species. Pore size distribution analysis further demonstrated that **Co_3_S_4_@Co‐BPDC‐3** possesses a hierarchical mesoporous structure with dominant pore sizes predominantly ranging from 5 to 20 nm (Figure S4b).

### 2.3 Electrochemical Properties of Nanosheet **Co_3_S_4_@Co‐BPDC‐3**

**FIGURE 5:**
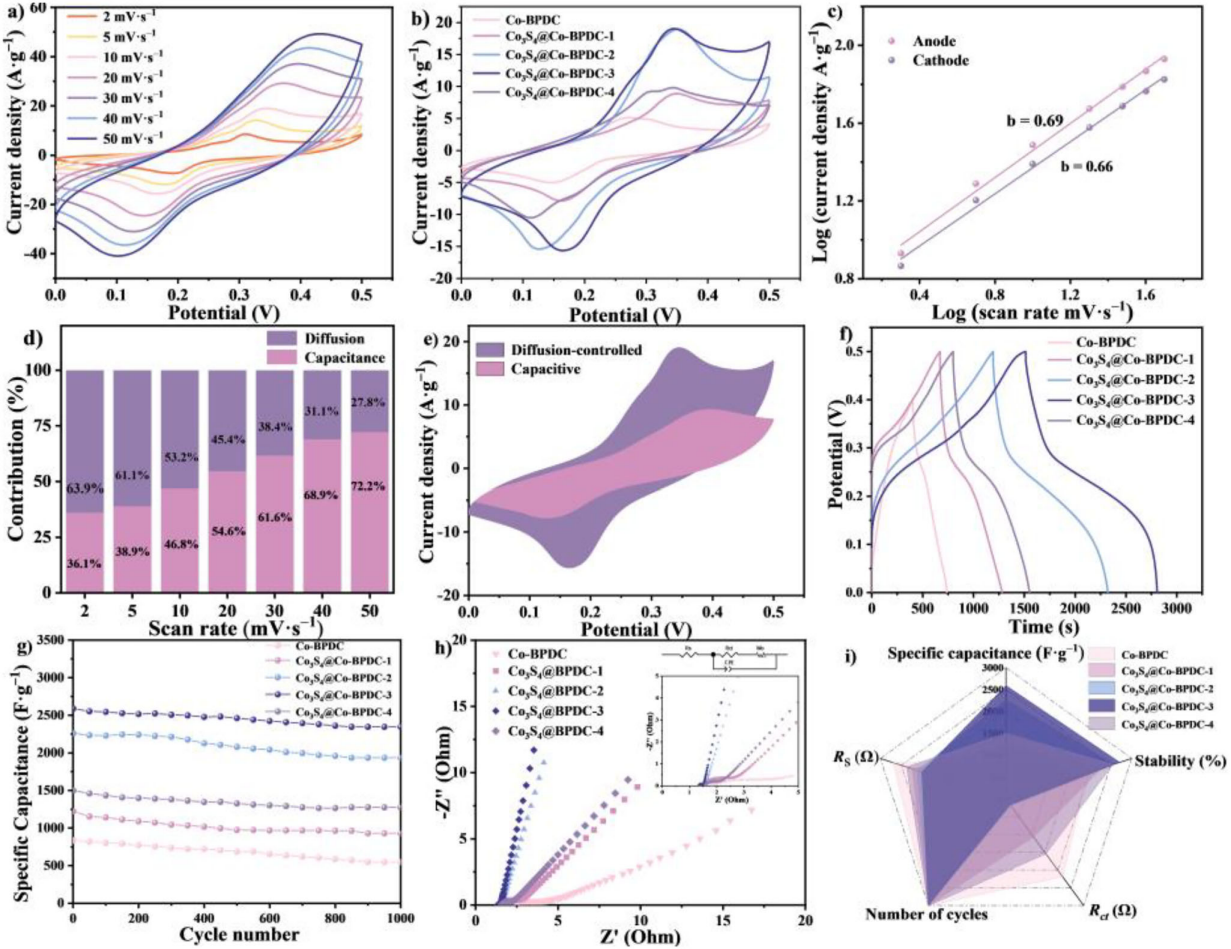
(a) CV curves of **Co_3_S_4_@Co‐BPDC‐3** at varying scan rates of ‌2–50 mV·s^−1^‌. (b) CV curves of the electrodes at a scan rate of ‌10 mV·s^−1^. (c) Relationship between the peak current (*i*) and scan rate (*v*) for **Co_3_S_4_@Co‐BPDC‐3**. (d) Variation of pseudocapacitive contribution percentage for **Co_3_S_4_@Co‐BPDC‐3** at different scan rates. (e) Separation of the diffusion and capacitive‐controlled currents of the electrode **Co_3_S_4_@Co‐BPDC‐3** at a scan rate of 10 mV·s^−1^. (f) GCD profiles of the electrodes at a current density of ‌1 A·g^−1^. (g) Cycling stability tests at a high current density of ‌10 A·g^−1^. (h) EIS Nyquist plots. (i) Comparative electrochemical performance metrics of electrodes **Co‐BPDC** and **Co_3_S_4_@Co‐BPDC‐1** to **Co_3_S_4_@Co‐BPDC‐4**.

To validate the electrochemical advantages of as‐synthesized sulfurized nanosheets, this study comparatively tested samples both before and after sulfurization as cathode materials. To demonstrate the superior electrochemical performance of the composite nanosheets, **Co_3_S_4_@Co‐BPDC‐1** to **Co_3_S_4_@Co‐BPDC‐4**, and **Co‐BPDC** were prepared with mass loadings of 5.2, 5.1, 5.2, 5.3, and 5.0 mg, respectively. The electrochemical behavior was comprehensively assessed in a three‐electrode system with 1.0 M KOH electrolyte through characterization techniques including cyclic voltammetry (CV), galvanostatic charge‐discharge (GCD), electrochemical impedance spectroscopy (EIS), and long‐term stability measurements. The CV curves of the **Co_3_S_4_@Co‐BPDC‐3** electrode and other electrodes at scan rates from 2 to 50 mV·s^−1^ are depicted in Figure 5a and Figure S5, Distinct redox peaks are evident in all samples, indicating significant pseudocapacitive behavior. The oxidation and reduction peaks correspond to the reversible redox reaction for Co^2+^/Co^3+^. The various oxidation states of cobalt and the charge‐discharge mechanism may be probably described by the following Equations (1) and (2) (s and ad stand for the solid state and adsorption state, respectively) [37]:

(1) $$Co^{\mathrm{II}}S_{s}+OH^{-}⇌Co^{\mathrm{II}}S_{s}{\left(\mathrm{OH}\right)}_{\mathrm{ad}\;}+e^{-}$$

(2) $$Co^{\mathrm{II}}S_{s}{\left(\mathrm{OH}\right)}_{\mathrm{ad}}⇌Co^{\mathrm{III}}S_{s}{\left(\mathrm{OH}\right)}_{\mathrm{ad}}+e^{-}$$

Furthermore, CV curves of electrodes **Co_3_S_4_@Co‐BPDC and Co‐BPDC** at a scanning rate of 10 mV·s^−1^ within a voltage range of 0 to 0.5 V are shown in Figure 5b. Compared with the **Co‐BPDC** electrode, the **Co_3_S_4_@Co‐BPDC** electrodes exhibit higher peak current at redox potentials and a larger enclosed area in the CV curves, and it is known that the specific capacity is directly proportional to the enclosed area of the CV curve, so **Co_3_S_4_@Co‐BPDC** electrodes demonstrated increased specific capacities after sulfidation. This significant improvement likely stems from the morphological evolution of the sulfurized samples during synthesis, wherein the linear precursor transforms into a petal‐like structure, exposing more active sites while the MOF‐derived sulfides enhance the structural stability of the composite nanosheet [38].

Based on the analysis of curves at the scanning rates of 2–50 mV·s^−1^, the charge storage mechanism and reaction kinetics of **Co_3_S_4_@Co‐BPDC‐3** were elucidated. The charge storage behavior of the composite was investigated by examining the relationship between peak current (*i*) and scan rate (*v*), where linear dependencies of anodic/cathodic peak currents versus scan rate were established as Formula S1. Typically, the charge storage mechanism in electrode **Co_3_S_4_@Co‐BPDC‐3** involves a synergistic interplay between the surface capacitance and diffusion control, corresponding to *b*‐values ranging from 0.5 to 1.0 [39]. As shown in Figure 5c, the *b*‐values of electrode **Co_3_S_4_@Co‐BPDC‐3** are 0.69 and 0.66 at different peak currents, confirming a hybrid charge storage mechanism, where the charge storage process in the composite material arises from the concerted contribution of both surface capacitive effects and diffusion‐controlled processes. The relative contributions of surface‐capacitive and diffusion‐controlled processes can be quantitatively determined using Formula S2. Figure 5d quantifies the percentage contributions of surface‐capacitive versus diffusion‐controlled mechanisms across varying scan rates, demonstrating a progressive increase in capacitive dominance with escalating scan rates. Complementary capacitive contribution profiles for **Co_3_S_4_@Co‐BPDC‐3** at scan rates spanning 2–50 mV·s^−1^ are presented in Figure 5e and Figure S6. Quantitative analysis reveals that the surface‐capacitive contribution to total charge storage increases substantially from 36.1% to 72.2%, while diffusion‐controlled participation concomitantly declines from 63.9% to 27.8%. This inverse relationship unequivocally demonstrates the dominant role of rapid interfacial Faradaic redox reactions during electrochemical charge/discharge processes.

Figure S7 depicts the GCD curves for electrodes **Co_3_S_4_@Co‐BPDC** and **Co‐BPDC** at varying current densities of 1–10 A·g^−1^ within the potential range of 0–0.5 and 0–0.4 V, respectively, in which the distinct voltage plateaus and the charge‐discharge curves approaching symmetry further confirm the typical pseudocapacitive behavior, in agreement with the results of CV. The electrodes **Co_3_S_4_@Co‐BPDC** demonstrate much better charge storage capability than that of **Co‐BPDC**, especially for **Co_3_S_4_@Co‐BPDC‐3**, which gives the longest discharge time (Table 1). The comparison of GCD curves of the electrodes at the current density of 1 A·g^−1^ is depicted in Figure 5f, in which the discharge times of electrodes **Co_3_S_4_@Co‐BPDC** are 611.2, 1130.3, 1294.6, and 749.2 s, but 334.5 s for **Co‐BPDC**. According to Formula S4, it can be calculated that the specific capacitance of the electrode **Co_3_S_4_@Co‐BPDC‐3** has reached a significant value of 2589.2 F·g^−1^ at 1 A·g^−1^. It is noteworthy that the maximum test voltage for electrode **Co‐BPDC** is limited to 0.4 V, whereas electrodes **Co_3_S_4_@Co‐BPDC** exhibit a higher voltage threshold of 0.5 V. This discrepancy may stem from the lamellar morphology of **Co_3_S_4_@Co‐BPDC**, which can exhibit a more stable electrode system as mentioned above. In addition, the interlayer gaps facilitate electrolyte penetration and ion transport, and thereby a lower charge transfer resistance (*R*_ct_) of the **Co_3_S_4_@Co‐BPDC** electrode enables operation at higher potentials without side reactions (vide infra). Furthermore, electrode **Co_3_S_4_@Co‐BPDC‐3** exhibits exceptional cycling stability, with its performance remaining essentially unchanged after 800 cycles and achieving 90.5% capacitance retention after 1000 cycles at 10 A g^−1^, outperforming all other counterparts (Figure 5g). The typical GCD curves of the **Co_3_S_4_@Co‐BPDC‐3** composite nanosheet are presented for the first three cycles and the last three cycles (the inset of Figure S8a). The similarity in the time‐potential response behavior of each charge‐discharge cycle indicates that each cycle process is reversible and the electrode material is stable. The outstanding rate capability and cycle life are ascribed to its robust two‐dimensional porous framework, which ensures efficient ion‐transport pathways even under high‐current‐density operation [40]. Through electrochemical impedance spectroscopy (EIS) and equivalent circuit fitting, a detailed analysis of charge transfer and electrolyte diffusion processes was conducted (Figure 5h). The results demonstrate that **Co_3_S_4_@Co‐BPDC‐3** exhibits a significantly reduced charge transfer resistance (*R*_ct_ = 0.16 Ω), far lower than that of the comparative samples: **Co‐BPDC** (2.19 Ω), **Co_3_S_4_@Co‐BPDC‐1** (1.54 Ω), **Co_3_S_4_@Co‐BPDC‐2** (0.18 Ω), and **Co_3_S_4_@Co‐BPDC‐4** (1.06 Ω). In addition, the near‐vertical slope of the **Co_3_S_4_@Co‐BPDC‐3** curve in the low‐frequency region indicates rapid ion diffusion and deep penetration. This outstanding electrochemical performance can be attributed to the unique petal‐like nanosheet structure of **Co_3_S_4_@Co‐BPDC‐3**, which not only provides a larger electroactive surface area but also creates abundant electrolyte‐accessible sites, thereby significantly enhancing charge transfer efficiency. Additionally, the radar chart in Figure 5i visually demonstrates the superior electrochemical performance of the electrode **Co_3_S_4_@Co‐BPDC‐3** across all evaluated metrics compared to all other electrodes. This comprehensive outperformance provides a quantitative validation of our structural design hypothesis. Moreover, **Co_3_S_4_@Co‐BPDC‐3** was further characterized by FT‐IR, XRD, and EIS after 1000 charge‐discharge cycles. The FT‐IR absorption peaks remained largely unchanged (Figure S8b), confirming the preservation of the chemical structure and coordination environment during prolonged cycling. The slight peak shifts are attributable to lattice strain, local environmental changes from repeated ion intercalation/deintercalation, and possible minor variations in cobalt oxidation state. XRD analysis revealed no significant phase transformation, only a slight decrease in peak intensity and a minor peak shift (Figure S8c), likely due to repeated ion insertion/extraction that may marginally reduce crystallinity over long‐term cycling. Moreover, **Co_3_S_4_@Co‐BPDC‐3** exhibits excellent electrochemical stability, with its *R*_ct_ impedance increasing only slightly from 0.16 to 0.18 and the ion‐diffusion‐related sloping line remaining well‐maintained, showing only a weak decreasing trend (Figure S8d). Overall, the FT‐IR, XRD, and EIS results collectively show that the **Co_3_S_4_@Co‐BPDC‐3** electrode remains structurally and chemically stable after 1000 charge‐discharge cycles.

**TABLE 1:** Discharge times and specific capacitances for electrodes based on **Co_3_S_4_@Co‐BPDC** and **Co‐BPDC** with current densities of 1, 2, 5 and 10 A·g^−1^. (Note: **1**–**4** correspond to **Co_3_S_4_@Co‐BPDC‐1** to **Co_3_S_4_@Co‐BPDC‐4**, respectively.)

Current Density (A·g^−1^)	Discharge Time (s)					Specific Capacitance (F·g^−1^)				
	Co_3_S_4_@Co‐BPDC				Co‐BPDC	Co_3_S_4_@Co‐BPDC				Co‐BPDC
	**1**	**2**	**3**	**4**		**1**	**2**	**3**	**4**	
1	611.2	1130.3	1294.6	749.2	334.5	1222.4	2260.6	2589.2	1489.4	836.3
2	346.8	466.3	605.9	356.2	150.3	1387.2	1865.2	2423.6	1424.8	751.5
5	96.2	182.5	199.7	150.4	44.3	962.0	1825.0	1997.2	1504.2	553.8
10	31.2	67.2	83.0	44.4	19.3	624.0	1344.0	1660.2	888.0	482.5

**FIGURE 6:**
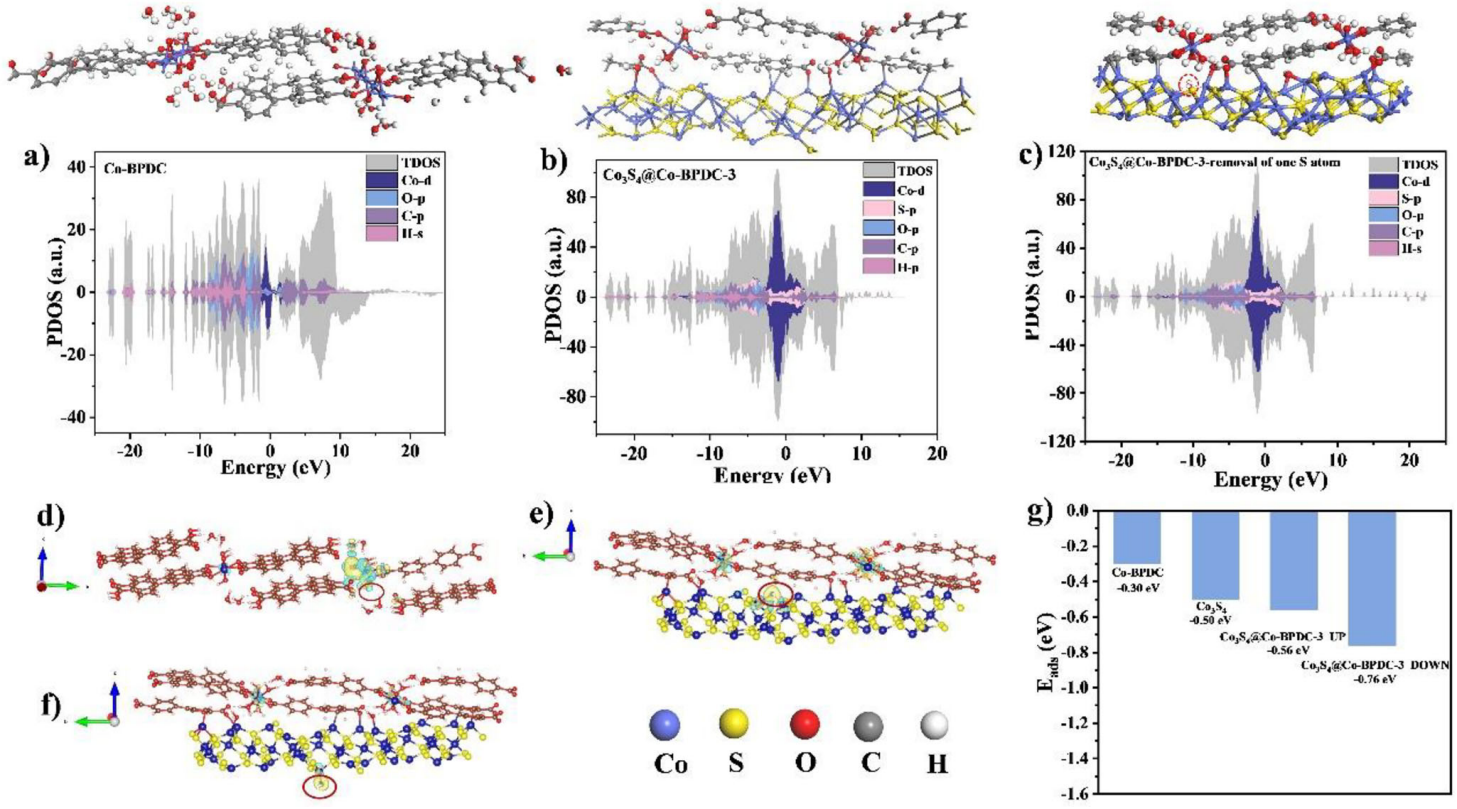
Schematic electronic structures with the corresponding PDOS profiles for (a) **Co‐BPDC**; (b) **Co_3_S_4_@Co‐BPDC‐3** and (c) **Co_3_S_4_@Co‐BPDC‐3**‐removal of one S atom (the red dashed circle marks the location of the introduced S vacancy), respectively. (d–f) Differential charge density maps and charge transfer analysis of OH* adsorbed on metal sites. (g) Adsorption energy of OH* adsorbed on the metal sites of Co_3_S_4_; **Co‐BPDC** and **Co_3_S_4_@Co‐BPDC‐3** heterojunction up and down.

To thoroughly investigate the enhancing effect of sulfurization treatment on material performance, this study systematically compared and analyzed the electronic structural characteristics of electrode materials before and after sulfurization based on DFT calculations. The optimized crystal structures of **Co‐BPDC** and **Co_3_S_4_@Co‐BPDC‐3** were systematically established, followed by the calculations of their respective projected density of states (PDOS) distributions, with results presented in Figure 6a,b. **Co_3_S_4_@Co‐BPDC‐3** exhibits significantly enhanced PDOS peaks near the Fermi level, demonstrating stronger orbital hybridization, pronounced electron accumulation effects, and superior electron transfer efficiency. Notably, the PDOS in both materials predominantly originates from the *d*‐orbitals of Co atoms, thereby demonstrating the dominant role of transition metals in governing electronic structures. We also introduced S vacancies at the interface (Figure 6c). The PDOS analysis of the defective heterojunction reveals that the total density of states (TDOS) at the Fermi level remains at a relatively high non‐zero value, indicating that the metallic characteristics are well preserved. The Co‐3d orbitals continue to dominate the electronic states near the Fermi level. Importantly, the introduction of vacancies does not generate localized defect states within the band gap, nor does it significantly reduce the density of states at the Fermi level. These results suggest that the electronic conducting channels in the heterojunction are primarily provided by the Co sites, and the removal of interfacial S atoms does not disrupt the core pathways for electron transport. Consequently, the system maintains excellent electronic conductivity even in the presence of defects. Through systematic modulation of distinct adsorption site configurations, all OH^−^ species were unambiguously confirmed to preferentially adsorb onto surface‐layer cobalt atoms (as corroborated by Figure S9). Bader charge analysis reveals charge transfer magnitudes at the metal sites of **Co‐BPDC** and **Co_3_S_4_@Co‐BPDC‐3** heterojunction in the up and down configurations are 0.37, 0.52, and 0.41 eV, respectively (Table S2). Differential charge density analysis further elucidated the electronic interaction mechanism during OH* adsorption (Figure 6d–f): significant orbital hybridization occurs between the *p*‐orbitals of oxygen atoms and *d*‐orbitals of Co atoms, facilitating electron transfer that reconstructs the electron density distribution around Co centers [41]. This electronic redistribution ultimately results in the formation of new chemical bonds between O and Co atoms.

The adsorption energetics of OH* in alkaline electrolyte environments were further investigated, with detailed results presented in Figure 6g and Table S3. The computed adsorption energies for **Co‐BPDC**, Co_3_S_4_, **Co_3_S_4_@Co‐BPDC** heterojunction in the up and down configurations are −0.30, −0.50, −0.56, and −0.76 eV, respectively. The significantly lower adsorption energy exhibited by the **Co_3_S_4_@Co‐BPDC**‐down heterojunction configuration indicates superior OH* binding affinity [42]. The enhanced adsorption properties of **Co_3_S_4_@Co‐BPDC** significantly accelerate its reaction kinetics in alkaline media, thereby providing a theoretical basis for the material's exceptional electrochemical performance.

Based on the above calculations and analyses, it can be concluded that the sulfurization treatment could induce synergistic interactions between metal sulfides and the MOF framework, effectively activating metal active sites and significantly enhancing the charge transfer capacity of Co centers. This results in a substantial increase in the material's total electronic PDOS. These theoretical findings are highly consistent with our experimental results, providing robust mechanistic insights into performance enhancement.

### 2.4 Electrochemical Performance of **Co_3_S_4_@Co‐BPDC‐3**//AC Based ASC

**FIGURE 7:**
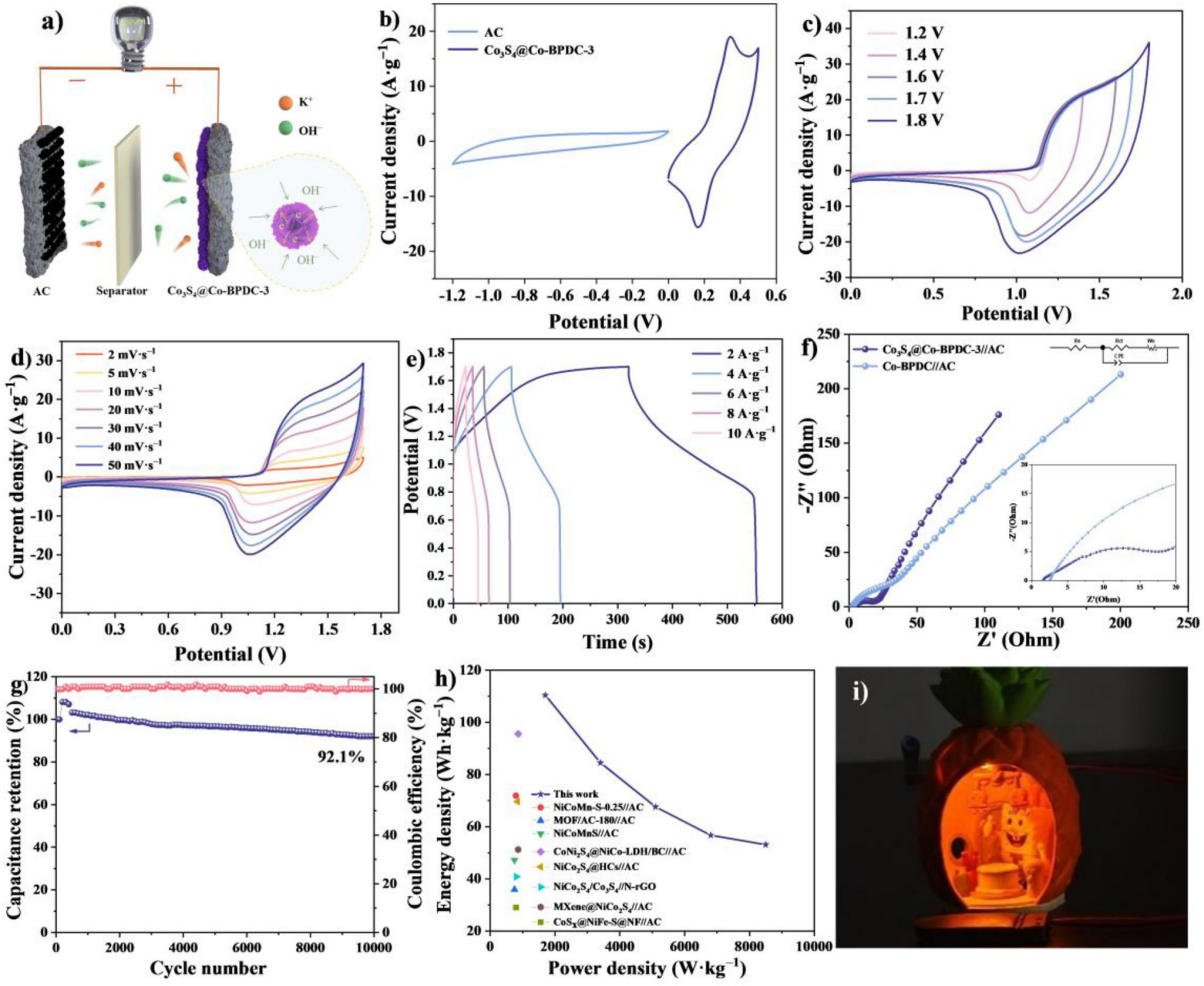
(a) Schematic diagram of the **Co_3_S_4_@Co‐BPDC‐3**//AC model. (b) CV curves of **Co_3_S_4_@Co‐BPDC‐3** and AC at a scan rate of 10 mV·s^−1^. (c) CV curves of **Co_3_S_4_@Co‐BPDC‐3**//AC at different voltage windows under a scan rate of 50 mV·s^−1^. (d) CV curves of **Co_3_S_4_@Co‐BPDC‐3**//AC based device at scan rates ranging from 2 to 50 mV·s^−1^. (e) GCD curves of **Co_3_S_4_@Co‐BPDC‐3**//AC based device. (f) EIS plots of **Co_3_S_4_@Co‐BPDC‐3**//AC and **Co‐BPDC**//AC based devices (with insets showing their magnified views and equivalent circuit diagrams). (g) Cycling stability and coulombic efficiency of **Co_3_S_4_@Co‐BPDC‐3**//AC based device at the current density of 10 A·g^−1^ after 10 000 cycles. (h) Ragone plot comparison of **Co_3_S_4_@Co‐BPDC‐3**//AC with previously reported similar supercapacitors. (i) Demonstration of **Co_3_S_4_@Co‐BPDC‐3**//AC based device powering an OLED.

Based on the electrochemical analysis confirming the superior performance of **Co_3_S_4_@Co‐BPDC‐3**, a coin‐type ASC was fabricated with **Co_3_S_4_@Co‐BPDC‐3** as the cathode, AC as the anode, and 6 mol·L^−1^ KOH as the electrolyte (Figure 7). Additionally, **Co‐BPDC**//AC was ‌also fabricated‌ using **Co‐BPDC** as the cathode for comparative purposes‌ (Figure S10). The device structure is schematically illustrated in Figure 7a. CV curves obtained at a scan rate of 10 mV·s^−1^ demonstrate matched curve geometries between AC and **Co_3_S_4_@Co‐BPDC‐3**, confirming their compatibility for effective voltage window extension (Figure 7b). Notably, at 50 mV·s^−1^, the **Co_3_S_4_@Co‐BPDC‐3**//AC assembly demonstrates an expanded operational voltage window from 1.2 to 1.8 V (Figure 7c). Both CV curves across varied scan rates (0–1.7 V) of **Co_3_S_4_@Co‐BPDC‐3**//AC and **Co‐BPDC**//AC based device exhibit quasi‐rectangular shapes with pronounced redox peaks, indicative of a hybrid charge‐storage mechanism synergizing double‐layer capacitance and pseudocapacitance (Figure 7d and Figure S10a). Critically, the well‐preserved CV profiles under escalating scan rates and potential windows robustly demonstrate exceptional redox reversibility and rate capability [43]. The GCD profiles of **Co_3_S_4_@Co‐BPDC‐3**//AC based device deliver specific capacitances of 275.1, 210.4, 168.4, 141.2, and 132.4 F·g^−1^ at current densities of 2, 4, 6, 8, and 10 A·g^−1^, respectively, which are better than that of **Co‐BPDC**//AC based device, demonstrating outstanding rate capability (Figure 7e and Figure S10b and Table S4). EIS analysis reveals a significantly reduced semicircular diameter in the high‐frequency region for **Co_3_S_4_@Co‐BPDC‐3**//AC based device compared to **Co‐BPDC**//AC based device, indicating decreased *R*_ct_ and accelerated Faradaic kinetics (Figure 7f). The slope >45° in the low‐frequency region confirms rapid ion diffusion, while the low equivalent series resistance (1.37 Ω) facilitates efficient charge transport. As shown is Figure 7g, the cycling tests of **Co_3_S_4_@Co‐BPDC‐3**//AC based device exhibit 92.1% capacitance retention with nearly 100% coulombic efficiency after 10 000 cycles at 10 A·g^−1^ in the potential range of 0–1.7 V, whereas the **‌Co‐BPDC**//AC‌ based device only achieves ‌81.6%‌ (Figure S10c). During the initial 1800 cycles, the **Co_3_S_4_@Co‐BPDC‐3**//AC based device undergoes activation, with the specific capacitance exhibiting a significant upward trend, reaching a maximum of 108.1% of the initial specific capacitance. According to Formulas S5 and S6, the **Co_3_S_4_@Co‐BPDC‐3**//AC based device achieves an energy density of 110.4 Wh·kg^−1^ with a power density of 1699.9 W·kg^−^^1^ at the current density of 2 A·g^−1^, retaining 53.1 Wh·kg^−^^1^ at 8500.0 W·kg^−1^ when the current density is up to 10 A·g^−1^. Its performance not only significantly surpasses that of the **Co‐BPDC**//AC based device but also outperforms those of most other reported sulfur‐containing ASC electrode materials, for instance, CoS_x_@NiFe‐S@NF//AC [44], NiCo_2_S_4_/Co_3_S_4_//N‐rGO [45], Ni_9_S_8_@C‐5//CTP‐AC [46], CoNi_2_S_4_@NiCo‐LDH/BC//AC [47], MXene@NiCo_2_S_4_//AC [48], NiCoMnS//AC [49], NiCo_2_S_4_@HCs//AC [33] and NiCoMn‐S‐0.25//AC [50] (Figure 7h and Table 2). Additionally, the practical feasibility is further validated by two tandem‐assembled ASC devices successfully powering an organic light‐emitting diode (OLED) for over 10 min, demonstrating the promising potential of **Co_3_S_4_@Co‐BPDC‐3** nanosheets for advanced energy storage systems (Figure 7i).

**TABLE 2:** Comparison of electrochemical performance of ASC devices assembled with different active materials for this work and previous reports.

Electrode materials	Energy density (Wh·kg^−1^)	Power density (W·kg^−1^)	Stability	Refs.
**Co_3_S_4_@Co‐BPDC‐3** //AC	110.4	1699.9	92.1% after 10 000 cycles at 10 A·g^−1^	This Work
CoS_x_@NiFe‐S@NF//AC	29	800	80.7% after 5000 cycles at 10 A·g^−1^	[44]
NiCo_2_S_4_/Co_3_S_4_//N‐rGO	40.8	806.3	89.6% after 5000 cycles at 10 A·g^−1^	[45]
Ni_9_S_8_@C‐5//CTP‐AC	69.32	800.06	83.06% after 5000 cycles at 5 A·g^−1^	[46]
CoNi_2_S_4_@NiCo‐LDH/BC//AC	95.57	866	95.2% after 10 000 cycles at 20 A·g^−1^	[47]
MXene@NiCo_2_S_4_//AC	51.21	863.65	90.7% after 10 000 cycles at 5 A·g^−1^	[48]
NiCoMnS//AC	47.2	750	85.2% after 10 000 cycles at 5 A·g^−1^	[49]
NiCo_2_S_4_@HCs//AC	69.6	847	90.2% after 10 000 cycles at 20 A·g^−1^	[33]
NiCoMn‐S‐0.25//AC	71.86	793.56	90.1% after 10 000 cycles at 10 A·g^−1^	[50]

### 2.5 Preparation and Electrochemical Performance of **Co_3_S_4_@Co‐BPDC‐3**//AC Based Flexible ASC

**FIGURE 8:**
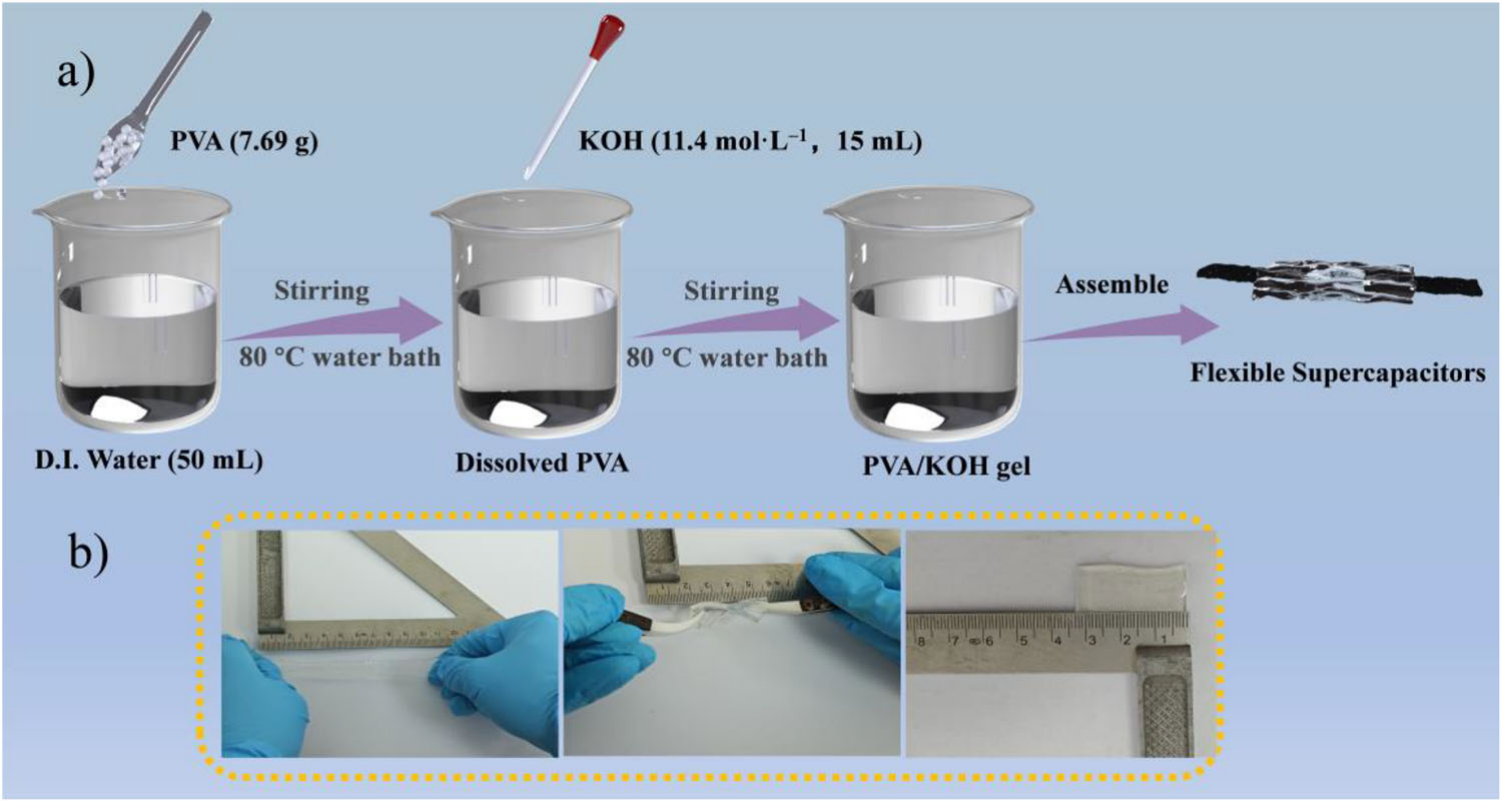
(a) Preparation route for the PVA/KOH gel and the assembly of **Co_3_S_4_@Co‐BPDC‐3**//AC based flexible device. (b) Photographs showing the PVA/KOH gel in its during stretching, under twisting, and original state.

To gain a deeper understanding of the real‐world applicability of the **Co_3_S_4_@Co‐BPDC‐3** electrode, **Co_3_S_4_@Co‐BPDC‐3**//AC based flexible ASC device was also constructed using PVA (polyvinyl alcohol)/KOH as the gel electrolyte. The preparation of PVA/KOH gel and the assembly of the flexible device are schematically illustrated in Figure 8a, and the detailed synthesis procedures are available in the supporting information. As demonstrated in Figure 8b, the prepared PVA/KOH gel exhibits superior flexibility while maintaining structural integrity under mechanical deformations including twisting, stretching, and compression [51].

**FIGURE 9:**
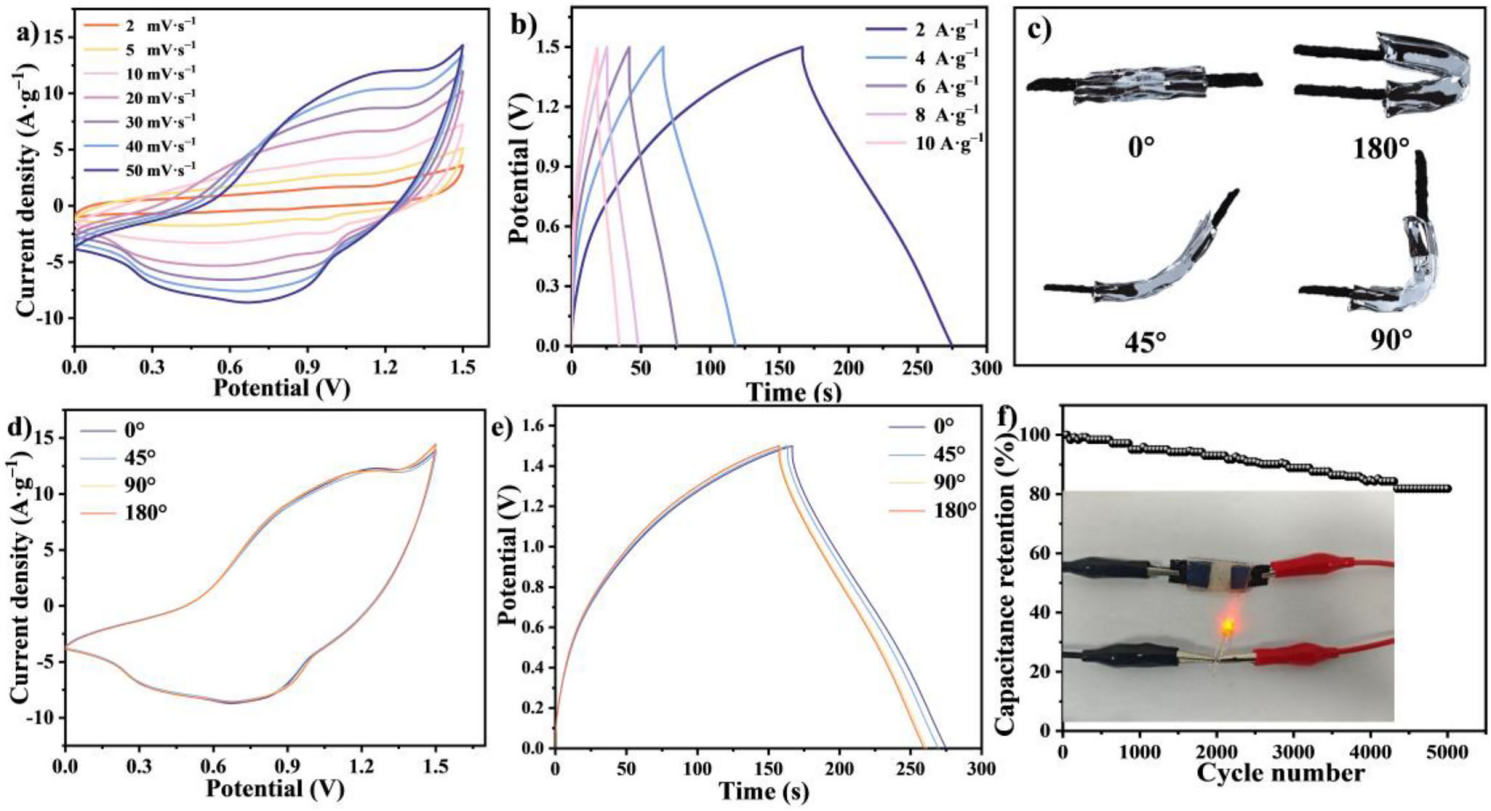
(a) CV curves at scan rates of 2–50 mV·s^−1^ for the as‐prepared flexible ASC device. (b) GCD curves at current densities of 2–10 A·g^−1^ for the as‐prepared flexible ASC device. (c) Schematic diagram illustrating the folding principles of the as‐prepared flexible ASC device at 0°, 45°, 90°, and 180°. (d) CV curves of the as‐prepared flexible ASC device at different bending angles. (e) GCD curves of the as‐prepared flexible ASC device at different bending angles. (f) Cycling stability of the as‐prepared flexible ASC device at the current density of 10 A·g^−1^ after 5000 cycles (Inset: Demonstration of the as‐prepared flexible ASC device successfully powering an OLED).

As shown in Figure 9a, CV curves of the as‐fabricated flexible device within 0–1.5 V at the scan rates of 2–50 mV·s^−1^ exhibit well‐retained quasi‐rectangular shapes at low scan rates, indicating ideal capacitive characteristics [52]. GCD profiles reveal that the as‐prepared flexible device yields specific capacitances of 144.3, 123.2, 104.0, 90.7, and 80.7 F·g^−1^ at the current densities of 2, 4, 6, 8, and 10 A·g^−1^, respectively (Figure 9b). As shown in Figure S11, the Nyquist plot demonstrates that the as‐fabricated flexible device exhibits a steep low‐frequency region slope (>45°), indicating accelerated ion diffusion kinetics. Along with this, the as‐fabricated flexible device shows an equivalent series resistance of 0.46 Ω, confirming reduced internal resistance conducive to rapid ion transport. Mechanical‐electrochemical stability validation via bending tests reveals exceptional performance (Figure 9c) [53]. CV curves remain nearly identical at the bending angles of 0°, 45°, 90°, and 180° (Figure 9d). The corresponding GCD profiles measured at 2 A·g^−^^1^ further confirm minimal capacitance loss under bending deformation, with retention rates of 98% (45°), 95.1% (90°), and 94.4% (180°), respectively. This superior bending tolerance originates from efficient interfacial stress transfer between the gel electrolyte and electrode during mechanical deformation.‌ ‌As shown in Figure 9f, the as‐fabricated flexible device demonstrates outstanding electrochemical stability throughout 5000 GCD cycles, maintaining 98.4% capacitance retention within the initial 600 cycles and 84.8% retention after 4000 cycles. Subsequently, the capacitance decreases to 81.8%. At 2 A·g^−1^, the as‐fabricated flexible device achieves energy density of 45.1 Wh·kg^−1^ with a power density of 1500.3 W·kg^−1^, while maintaining 25.2 Wh·kg^−1^ with a power density of 7500.0 W·kg^−1^ when operated at 10 A·g^−1^ (Table S5). The practical applicability is unequivocally demonstrated through successful powering of an OLED (Figure 9f), showcasing the device's promising real‐world performance.

## 3 Conclusion

By a MOF‐templated synthesis method, a series of novel petal‐like 2D **Co_3_S_4_@Co‐BPDC** composite nanosheets have been successfully synthesized. Specifically, **Co‐BPDC** was used as a sacrificial template to be sulfurized with thiourea by an in situ hydrothermal growth to give the as‐prepared nanosheets. By adjusting the dosage of the thiourea, the morphology of the composite materials can be tuned to achieve optimal petal‐like 2D nanostructures, which have been verified by SEM, TEM, and HRTEM. Combining the advantages of MOFs and metal sulfides, **Co_3_S_4_@Co‐BPDC** nanosheets have good stability, which has been identified by XRD, SEM/EDX, and XPS. The resulting **Co_3_S_4_@Co‐BPDC‐3** electrode without binders and conductive additives delivers an exceptional specific capacitance of 2589.2 F·g^−1^ at 1.0 A·g^−1^, coupled with an outstanding cycling stability (90.5% capacitance retention after 1000 cycles). DFT calculations were conducted and confirmed that this sulfurization significantly enhanced the OH* adsorption capability and electron transfer kinetics of the **Co_3_S_4_@Co‐BPDC‐3** nanosheet, demonstrating stronger orbital hybridization, pronounced electron accumulation effects, and superior electron transfer efficiency. Furthermore, both the coin‐type ASC device and flexible ASC device were fabricated and exhibited excellent electrochemical energy storage properties. The former demonstrates a maximum energy density of 110.4 Wh·kg^−1^ at a power density of 1699.9 W·kg^−1^ and maintains 92.1% capacity retention after 10 000 cycles, while the latter gives a maximum energy density of 45.1 Wh·kg^−1^ at a power density of 1500.3 W·kg^−1^ and maintains 81.8% capacity retention after 5000 cycles. The flexible ASC device maintains 94.4% of its specific capacity even after 180° bending without structural failure. This work established a MOF‐templated synthesis method for 2D composite nanosheets, enabling a rational design strategy toward high‐performance 2D energy storage materials.

## Conflicts of Interest

The authors declare no conflicts of interest.

## Supporting information




**Supporting File**: smtd70839‐sup‐0001‐SuppMat.docx.

## Data Availability

The data that support the findings of this study are available in the supplementary material of this article.
