# Supplementary material for: Metal‐Organic Framework‐Templated Synthesis of 2D Co3S4@Co‐BPDC Nanosheets for High‐Performance Electrochemical Energy Storage
Source: Small Methods. 2026 Jul 14;10(15):e70839. doi: 10.1002/smtd.70839 (PMC13450283; doi:10.1002/smtd.70839)
Supplement: Supplementary file 1 [file SMTD-10-e70839-s001.docx]

Supporting Information

**Metal-Organic Framework-Templated Synthesis of 2D Co_3_S_4_@Co-BPDC Nanosheets for High-Performance Electrochemical Energy Storage**

Zhiwei Xu^1^, Qian Liu^1^***, Lin Zhou^1^, Mengru Huang^1^, Yanping Li^1^, Long Kuai^1^**,* Chuanjian Zhou^2^, Lijie Zhang^3^, Wai-Yeung Wong^4,5^*

Z. Xu, Q. Liu, L. Zhou, M. Huang, Y. Li, L. Kuai

^1^Anhui Province Key Laboratory of Functional Coordinated Complexes for Materials Chemistry and Application, School of Chemical and Environmental Engineering, Anhui Polytechnic University, Wuhu 241000, P.R. China

E-mail: lqcz_2008@ahpu.edu.cn, [kuailong@ahpu.edu.cn](mailto:kuailong@ahpu.edu.cn)

C. Zhou

^2^School of Material Science and Engineering and State Key Laboratory of Coatings for Advanced Equipment, Shandong University, Jinan 250071, P.R. China

L. Zhang

^3^Zhejiang International Joint Laboratory of Optical Functional Materials, Wenzhou University, Wenzhou 325000, P.R. China

W.-Y. Wong

^4^Department of Applied Biology and Chemical Technology and Research Institute for Smart Energy, The Hong Kong Polytechnic University, Hung Hom, Kowloon, Hong Kong, P.R. China

^5^The Hong Kong Polytechnic University Shenzhen Research Institute, Shenzhen 518057, P.R. China

E-mail: wai-yeung.wong@polyu.edu.hk

**1. Experimental section**

**1.1 Chemicals and reagents**

All chemical reagents were analytically pure and used directly without purification or treatment. Cobalt(II) nitrate hexahydrate (Co(NO_3_)_2_·6H_2_O, > 99.0%), ethanol (CH_3_CH_2_OH, AR, 95%), *N,N*-dimethylformamide (DMF, AR, ≥ 99.9%), thiourea (CS(NH_2_)_2_, AR, 99%), polyvinyl alcohol (PVA, AR, 99%) potassium hydroxide (KOH, 95%), and biphenyl-4,4’-dicarboxylic acid (BPDC, 98%) were sourced from Shanghai Titan Scientific Co., Ltd. The water used for the preparation of the solution was deionized (D.I.) water.

**1.2 Apparatus for characterization**

The morphology and dimensions of the fabricated materials were examined by utilizing a scanning electron microscope (SEM and EDX, Hitachi S-4800) and a field emission transmission electron microscopy (TEM and HRTEM, ThermoFischer FEI tecnai f20). At the same time, X-ray photoelectron spectroscopy (XPS) was conducted under 30 eV Al-Kα radiation using ThermoFischer Escalab Xi^+^. Fourier transform infrared spectroscopy (FT-IR) were recorded on the Shimadzu IR Prestige-21 FT-IR spectrometer using KBr pellets for solid state spectroscopy. Powder X-ray diffraction (PXRD) was performed on a Bruker D8 Advance X-ray diffractometer, with Cu Kα1 (45 kV, 100 mA). The Nitrogen adsorption-desorption measurements were conducted with an ASAP2460 Surface Area and Porosity Analyzer at 150 ℃.

**1.3 Electrochemical performance**

All electrochemical properties such as cyclic voltammetry (CV), galvanostatic charge-discharge (GCD), electrochemical impedance spectroscopy (EIS) and long-term stability measurements were performed in a three-electrode system containing 1.0 M KOH aqueous solution as the electrolyte on a CHI 660E electrochemical analyzer (CH Instruments, Chenhua. Co., Shanghai). The as-prepared **Co_3_S_4_@Co-BPDC** nanosheets were used as the working electrodes, a Hg/HgO and a platinum sheet were applied as the reference electrode and counter electrode, respectively. CV was measured within the potential window of 0~0.5 V at the scan rates of 2~50 mV·s^−1^. GCD curves were conducted in the potential range of 0~0.5 V at different constant current density with 1~10 A·g^−1^. The cycle life tests were carried out by GCD measurements with a constant current density of 10 A·g^−1^ for 1000 cycles. EIS measurements were performed in the AC frequency range of 10^−2^~10^5^ Hz with an amplitude of 5 V using a 1 M KOH electrolyte solution.

The relation of peak current (*i*) and sweep rate (*v*) can be described by the following formulas:^[1,2]^

*i* = *av^b^*	(S1)

log(*i*) = *b*log(*v*) + log(*a*)	(S2)

where *a* and *b* are adjustable parameters. The value of *b* can be calculated from the slope of log(*v*) vs log(*i*). Generally, the value *b* = 0.5 indicates a diffusion-controlled process, while the value *b* = 1 implies a typical capacitive contribution.

In addition, the capacitance contribution at different scanning rates can be derived from the following formula:

*i*(*V*) = *k*_1_*v* + *k*_2_*v*^1/2^	(S3)

where *k*_1_*v* and *k*_2_*v*^1/2^ reflect the surface-controlled process and diffusion-controlled process contribution, respectively.

The specific capacitance (*C*_s_) of the electrode material was derived from the GCD curve by using the following formula:^[3]^

 (S4)

Here, *C*_s_ is the specific capacitance (F·g^−1^), Δ*t* is the discharge time (s), *i*_m_ is the current density (A·g^−1^) and Δ*V* is the potential window (V).

**1.4 Fabrication and characterization of** **Co_3_S_4_@Co-BPDC-3//AC (active carbon) based asymmetric supercapacitor (ASC)**

For evaluating the application prospect of the electrode, asymmetric supercapacitor (ASC) should be prepared. An asymmetric supercapacitor consists of the **Co_3_S_4_@Co-BPDC-3** electrode, AC, and 6 M KOH aqueous solution, which was employed as the positive electrode, negative electrode, and electrolyte, respectively. The negative electrode was prepared for AC according to the literature methods as follows: the acquired AC, acetylene black, and PTFE were uniformly mixed in a mass ratio of 8:1:1, ground thoroughly, and coated onto nickel foam (NF) substrate. The mass ratio between electrodes was determined by balancing the stored charge in each electrode. Specifically, the charges stored in the positive (q⁺) and negative (q^−^) electrodes are calculated as q^+^ = C^+^ × ΔE^+^ × m^+^ and q^−^ = C^−^ × ΔE^−^ × m^−^, where C^+^/C^−^ represent the specific capacitances (F·g^−1^) of the positive/negative electrodes, ΔE^+^/ΔE^−^ denote their respective potential ranges (V), and m⁺/m⁻ correspond to the masses (g) of active material in each electrode. Charge balance is enforced through the equation q^+^ = q^−^. The supercapacitors were tested on Wuhan LanHe CT2001A test system.

The energy density (Wh·kg^−1^) and power density (W·kg^−1^) of the device were explored according to the following equations S5 and S6:^[4]^

$E=\frac{1}{2}\times\frac{C_{S}\times V^{2}}{3.6}$ (S5)

$P=\frac{3600E}{\Delta t}$ (S6)

where *E* is the energy density (Wh·kg^−1^), *P* is the power density (W·kg^−1^), *C_s_* is the specific capacitance (F·g^−1^), *V* is the voltage and Δ*t* is the discharge time (s).

**1.5 Testing of flexible supercapacitors using gel electrolytes**

For flexible supercapacitors, the as-prepared **Co_3_S_4_@Co-BPDC-3** and AC are employed as positive and negative electrodes, respectively. The preparation method of the negative electrode is consistent with that of the negative electrode for coin-type ASC device described in Section 1.4. 7.69 g of PVA was weighed and finely ground, then soaked in 50 mL of D.I. water. Subsequently, 6.39 g of 95% KOH was dissolved in 15 mL of D.I. water under vigorous stirring. The beaker containing the soaked PVA was placed in an 80°C water bath with continuous heating and stirring. After the solution thickened, the KOH solution was added dropwise into the PVA mixture. A layer of the PVA-KOH gel was then cast onto a plastic sheet, followed by assembly of the electrodes, and another layer of PVA gel was applied to encapsulate the device. The testing methodology aligns with the procedures described previously. The masses of the gel electrolyte and encapsulation materials are not included in the current calculation.

**1.6 Computational Details**

All the spin-polarized DFT calculations were performed using the projector augmented wave method (PAW)^[5]^ as implemented in the *Vienna Ab initio Simulation Package* (VASP)^[6]^. The generalized gradient approximation (GGA) with the Perdew-Burke-Ernzerhof functional (PBE)^[7]^ was used to describe the exchange-correlation interactions, and the van der Waals (vdW) correction proposed by Grimme (DFT-D3)^[8]^ was included in describing the long-range interactions. The cutoff energy of the plane-wave was set to 500 eV. All the structures were relaxed until the residual atomic forces on each atom were less than 0.02 eV/Å, and the total energy was less than 10^−5^ eV for self-consistent field (SCF) calculations. To improve the accuracy and efficiency, the Brillouin zone integration was performed on the 3 × 3 × 1 Monkhorst-Pack k-point mesh.^[9]^ The vacuum space was set to 15 Å in the z-direction to avoid interactions between periodic images.

|   **c)** |  |
| --- | --- |
|  | |

**Figure S1.** SEM images of a) **Co_3_S_4_@Co-BPDC-1**; b) **Co_3_S_4_@Co-BPDC-2** and c) **Co_3_S_4_@Co-BPDC-4**.

**b)**

**a)**

**Figure S2.** XRD patterns of **Co_3_S_4_@Co-BPDC-1**, **Co_3_S_4_@Co-BPDC-2** and **Co_3_S_4_@Co-BPDC-4**.

**b)**

**a)**

**Figure S3.** FT-IR spectra of BPDC, **Co-BPDC**, and **Co_3_S_4_@Co-BPDC-3.**

**Figure S4.** a) Nitrogen adsorption-desorption isotherms of **Co-BPDC** nanowires and **Co_3_S_4_@Co-BPDC-3** nanosheets. b) Pore size distribution curves of **Co-BPDC** and **Co_3_S_4_@Co-BPDC-3**.

**Figure S5.** CV curves of a) **Co-BPDC**; b) **Co_3_S_4_@Co-BPDC-1**; c) **Co_3_S_4_@Co-BPDC-2** and d) **Co_3_S_4_@Co-BPDC-4** with different thiourea additions in a three-electrode system at various scan rates.

**Figure S6.** Surface capacitive contribution plots of **Co_3_S_4_@Co-BPDC-3** at the scanning rate of a) 2 mV·s^−1^; b) 5 mV·s^−1^; c) 20 mV·s^−1^; d) 30 mV·s^−1^; e) 40 mV·s^−1^ and f) 50 mV·s^−1^.

**Figure S7.** GCD curves under the same voltage and different current densities of 1~10 A·g^−1^ in a three-electrode system for a) **Co-BPDC**; b) **Co_3_S_4_@Co-BPDC-1**; c) **Co_3_S_4_@Co-BPDC-2**; d) **Co_3_S_4_@Co-BPDC-3**; e) **Co_3_S_4_@Co-BPDC-4**; f) bare foamed nickel and g) GCD curves of AC electrode in three-electrode system at the current density of 5 A·g^−1^.

**Figure S8**. a) Cycling performance of the **Co_3_S_4_@Co-BPDC-3** electrode in the potential range of 0~0.5 V at 10 A·g^−1^ (with insets showing the GCD processes for the first three cycles and the last three cycles). Comparison of b) FT-IR; c) XRD patterns; and d) EIS for **Co_3_S_4_@Co-BPDC-3** before and after 1000 charge-discharge cycles.

**a)**

**b)**

**c)**

**Figure S9.** The schematic diagram of OH* adsorption on a) **Co-BPDC**; b) **Co_3_S_4_@Co-BPDC-3** heterojunction up and c) **Co_3_S_4_@Co-BPDC-3** heterojunction down.

**Figure S10.** a) CV curves of **Co-BPDC**//AC based device at scan rates ranging from 2 to 50 mV·s^−1^. b) GCD curves of **Co-BPDC**//AC ASC device. c) Cycling stability and coulombic efficiency of **Co-BPDC**//AC based device at a current density of 10 A·g^−1^ after 10000 cycles.

**Figure S11.** a) EIS Nyquist plots for **Co_3_S_4_@Co-BPDC-3**//AC based flexible device (Inset: The high-frequency region data). b) GCD profiles of flexible device devoid of active materials at the current densities of ‌2 and 4 A·g^−1^.

**Table S1.** Atomic percentage of elements in the XPS analysis of **Co_3_S_4_@Co-BPDC-3**.

| **Name** | **Atomic (%)** |
| --- | --- |
| C 1s | 52.38 |
| Co 2p | 7.31 |
| Ni 2p | 1.18 |
| O 1s | 32.19 |
| S 2p | 6.94 |

**Table S2.** Bader charge analysis for **Co-BPDC**, **Co_3_S_4_@Co-BPDC**-up and **Co_3_S_4_@Co-BPDC**-down, respectively.

| **Structure** | **slab** | **OH*** |
| --- | --- | --- |
| **Co-BPDC** | -0.37 | 0.37 |
| **Co_3_S_4_@Co-BPDC**-Up | -0.52 | 0.52 |
| **Co_3_S_4_@Co-BPDC**-Down | -0.41 | 0.41 |

**Table S3.** Adsorption energy of OH* on metal active sites of Co_3_S_4_, **Co-BPDC**, **Co_3_S_4_@Co-BPDC**-up and **Co_3_S_4_@Co-BPDC**-down, respectively.

| **Structure** | **Absorb OH* energy (eV)** |
| --- | --- |
| Co_3_S_4_ | −0.50 |
| **Co-BPDC** | −0.30 |
| **Co_3_S_4_@Co-BPDC**-up | −0.56 |
| **Co_3_S_4_@Co-BPDC**-down | −0.76 |

**Table S4.** Specific capacitance, energy density and power density of **Co-BPDC**//AC and **Co_3_S_4_@Co-BPDC-3**//AC devices at different current densities.

| Current density  (A·g^−1^) | Specific capacitance  (F·g^−1^) | | Energy density  (Wh·kg^−1^) | | Power density  (W·kg^−1^) | |
| --- | --- | --- | --- | --- | --- | --- |
|  | **Co-BPDC**  //AC | **Co_3_S_4_@Co-BPDC-3**//AC | **Co-BPDC**  //AC | **Co_3_S_4_@Co-BPDC-3**//AC | **Co-BPDC**  //AC | **Co_3_S_4_@Co-BPDC-3**//AC |
| 2 | 173.3 | 275.1 | 61.6 | 110.4 | 1600.5 | 1699.9 |
| 4 | 128.8 | 210.4 | 45.8 | 84.5 | 3200.0 | 3402.7 |
| 6 | 109.5 | 168.4 | 38.9 | 67.6 | 4800.0 | 5101.9 |
| 8 | 96.0 | 141.2 | 34.1 | 56.7 | 6400.0 | 6804.0 |
| 10 | 88.1 | 132.4 | 31.3 | 53.1 | 7999.5 | 8500.0 |

**Table S5.** Specific capacitance, energy density and power density of **Co_3_S_4_@Co-BPDC-3**//AC based flexible ASC device at different current densities.

| Current density  (A·g^−1^) | Specific capacitance  (F·g^−1^) | Energy density  (Wh·kg^−1^) | Power density  (W·kg^−1^) |
| --- | --- | --- | --- |
| 2 | 144.3 | 45.1 | 1500.3 |
| 4 | 123.2 | 38.5 | 2875.5 |
| 6 | 104.0 | 32.5 | 4500.0 |
| 8 | 90.7 | 28.3 | 6000.0 |
| 10 | 80.7 | 25.2 | 7500.0 |

**References**

[1] S. Wang, Y. Li, L. Wei, J. Zhu, Q. Zhang, L. Lan, L. Tang, F. Wang, Z. Zhang, L. Wang, J. Mao. Strain-insensitive supercapacitors for self-powered sensing textiles. *ACS Nano*, **2025**, *19*, 6357.
[2] D. Jia, K. Wang, X. Wang, Z. Zuo, X. Zhao, Q. Shen. Surface engineering of copper foam to construct a hierarchical heterostructure for high energy efficient supercapacitors. *Small*, **2024**, 2408572.
[3] Z. Wang, J. Chen, R. Bi, W. Dou, K. Wang, F. Mao, H. Wu, S. Wang. Supercapacitor and oxygen evolution reaction performances based on morphology-dependent Co-MOFs. *J. Solid State Chem.*, **2020**, *283*, 121128.
[4] X. Pu, X. Ren, H. Yin, Y. Tang, H. Yuan. One-step electrodeposition strategy for growing nickel cobalt hydroxysulfide nanosheets for supercapacitor application. *J. Alloys Compd.*, **2021**, *865*, 158736.
[5] G. Kresse, D. Joubert. From ultrasoft pseudopotentials to the projector augmented-wave method. *Phys. Rev. B*, **1999**, *59*, 1758.
[6] G. Kresse, J. Hafner. Ab initio molecular dynamics for liquid metals. *Phys. Rev. B*, **1993**, *47*, 558.
[7] G. Su, S. Yang, S. Li, C. J. Butch, S. N. Filimonov, J.-C. Ren, W. Liu. Switchable Schottky contacts: simultaneously enhanced output current and reduced leakage current. *J. Am. Chem. Soc.*‌‌, **2019**, *141*, 1628.
[8] S. Grimme, J. Antony, S. Ehrlich, H. Krieg. A consistent and accurate ab initio parametrization of density functional dispersion correction (DFT-D) for the 94 elements H-Pu. *‌J. Chem. Phys.,‌* **2010**, *132*, 154104.
[9] H. J. Monkhorst, J. D. Pack. Special points for Brillouin-zone integrations. *Phys. Rev. B*, **1976**, *13*, 5188.
